# The meta-analysis and systematic review of prevalence and clinical anatomy of the arc of Buhler

**DOI:** 10.1038/s41598-023-36316-9

**Published:** 2023-06-06

**Authors:** Katarzyna A. Kowalczyk, Jakub Pękala, Michał Kawzowicz, Przemysław A. Pękala, Krzysztof A. Tomaszewski

**Affiliations:** 1 Department of General and Oncological Surgery, 5th Military Clinical Hospital in Cracow, 30-901 Cracow, Poland; 2 Department of Anatomy, Jagiellonian University Medical College, 31-034 Cracow, Poland; 3 Faculty of Medicine and Health Sciences, Andrzej Frycz-Modrzewski Krakow University, 30-705 Cracow, Poland; 4 Jagiellonian University Medical College, 31-007 Cracow, Poland; 5 Scanmed St. Raphael Hospital, 30-693 Cracow, Poland

**Keywords:** Gastric cancer, Liver cancer, Pancreatic cancer, Embryology, Liver, Pancreas, Translational research

## Abstract

The arc of Buhler (AOB) is a direct anastomosis of the celiac axis and superior mesenteric artery. This paper reviews the literature on the AOB and provides accurate and up-to-date data on its prevalence, anatomy, and clinical significance. The main scholarly online databases were carefully searched for relevant studies related to the AOB. Information was gathered and formed the basis of the analysis of this study. In total, 11 studies were used in this meta-study, consisting of 3685 total patients tested and 50 cases of the AOB presented. The pooled prevalence estimate of the AOB was determined to be 1.7% (95% CI 0.9, 2.9). By imaging type, the prevalence of the AOB was 1.8% for radiological studies (n = 3485; 95% CI 0.9, 3.0), 1.4% for computed tomography (CT) studies (n = 1417; 95% CI 0.4, 3.0), and 1.9% for angiography studies (n = 2068; 95% CI 0.5, 4.0). The AOB is sufficiently significant and should be considered when planning surgeries or radiological procedures involving the abdomen.

## Introduction

The arc of Buhler (AOB) is a direct anastomosis of the celiac axis (CAx) and superior mesenteric artery (SMA). In addition to the AOB, there are two more types of anastomoses between the SMA and CAx described in the medical literature: between the gastroduodenal artery (CAx branch) and pancreaticoduodenal arteries (SMA branches) and between the dorsal pancreatic artery (CAx branch) and anterior/posterior pancreaticoduodenal arteries (SMA branches)^1^.

The AOB was first described in 1904 by Buhler^2^, who performed a series of dissections to establish the anastomotic variant, with a direct branch of the CAx forming a connection with a branch of the middle colic artery. Since its original discovery, the AOB has been mentioned numerous times in the scientific literature, usually described as a rare variant that is typically asymptomatic and is detected incidentally in patients who are undergoing imaging or abdominal surgery^3,4^.

The earliest account of embryology of the AOB is given by Tandler^5^, who proposed that the CAx and the SMA are derived from the 10th and 13th ventral segmental arteries, respectively, and that these arteries are connected by a prenatal ventral anastomosis that typically regresses^6^. A total or partial failure of regression will result in a persistent communicating artery between the CAx and SMA, resulting in an AOB. The AOB is rarely mentioned in modern anatomy texts and must usually be specifically searched for in the medical literature^6,7^.

The AOB runs a vertical retropancreatic course from the CAx to the SMA, usually directly connecting the main arteries, but it may also join more distal divisions^8,9^. For example, an exceptional case was reported by Olewnik^10^, in which the common hepatic artery formed an anastomosis with the SMA. The AOB typically runs a direct course, but there has been a reported case of the AOB giving off a small branch to the head of the pancreas^9^.

The presence of an AOB may help prevent mesenteric ischemia by allowing for collateral blood flow in the event of occlusion or compression of the CAx or SMA^11^. Surgical awareness of the SMA may help prevent unintentional iatrogenic injury during abdominal procedures^12^.

There is a significant gap in surgical education regarding the AOB, with only minor attention paid to vascular anatomical variations. This gap could result in a lack of awareness of potential harmful consequences during or after surgery. Our previous studies demonstrated that surgeons are generally not aware of the AOB’s function^13,14^. This knowledge deficit is clinically meaningful, as the AOB is not infrequent, with a reported prevalence of between 1 and 4% of the general population^6,15,16^. We undertook this systematic review and meta-analysis to review the literature on the AOB and provide accurate and up-to-date data on its prevalence, anatomy, and clinical significance.

## Methods

### Search strategy

The main scholarly online databases (Pubmed, Embase, ScienceDirect, and Web of Science) were carefully searched for relevant studies related to the AOB. The date and original language did not disqualify a study from inclusion. The databases were searched from their inception date until March 30, 2020. Upon collecting the relevant studies, a search was conducted to identify other potentially relevant studies that were not picked up during the original search. This study followed the Preferred Reporting Items for Systematic Reviews and Meta-Analyses (PRISMA).

### Eligibility assessment

The studies found during the searches were assessed for relevance by two independent reviewers. Studies were excluded if they (1) were individual case reports, (2) consisted of partial or difficult to extract data, or (3) were not conducted on humans. Some relevant studies were performed on patients with abdominal pathologies with the AOB as an incidental finding; these were included in this meta-analysis. Studies written in languages other than English were read by medical professionals proficient in the relevant language.

### Data extraction

For studies meeting the inclusion criteria, data extraction was performed separately by two researchers. Information regarding year, continent, country, type of study, imaging modality (computed tomography [CT] or angiography), number of subjects within the study, and number of patients with an AOB was gathered and formed the basis of the analysis of this study.

### Study endpoints

The purpose of the study was to determine the prevalence of the AOB among the general population. The morphology of the AOB in individual cases was not differentiated, and different anatomical variants were included in one general pool of data.

### Quality assessment

For the purpose of quality assessment and evaluating risk of study bias, the Anatomical Quality Assessment (AQUA) tool was used (http://www.eba.cm.uj.edu.pl/aqua-tool). Five separate domains were evaluated for risk of bias: (1) objectives and study characteristics, (2) study design, (3) methodology characterization, (4) descriptive anatomy, and (5) reporting of results. Using the AQUA tool, studies were evaluated based on a checklist of questions for each domain, with studies being assigned a high, low, or unclear risk of bias. Assessment of each domain ends with risk of bias questions, which are answered with yes, no, or unclear, indicating low, high, and unclear risk of bias, respectively. If all signaling questions for a domain are answered with yes, then the risk of bias can be judged as low. If any signaling question is answered with no, this indicates the potential for bias. In general, if a study does not contain a sufficient degree of information to assess the risk of bias within a domain, that domain is said to have an unclear level of risk. None of the studies assessed using the AQUA tool had an unclear level of risk for any domain.

The studies included in this meta-analysis revealed Domain 1 (objectives and subject characteristics) and Domain 3 (methodology characterization) to be at a high risk of bias, owing mainly to missing demographic data for the research group and no information regarding the researchers’ experience. Almost all studies had a low risk of bias for Domain 2 (study design), Domain 4 (descriptive anatomy), and Domain 5 (reporting of results). The results of the AQUA tool evaluation can be found in Table 1.

**Table 1:** Quality assessment of studies (using AQUA tool).

Study	Country	Type of study	Number of patients	Risk of bias—AQUA tool				
				Objectives and study characteristics	Study design	Methodology characterization	Descriptive anatomy	Reporting of results
Farghadani^17^	Iran	Radiological	607	High	Low	Low	Low	Low
McNulty^6^	Ireland	Radiological	300	High	Low	High	Low	Low
Sureka^18^	India	Radiological	600	Low	Low	Low	Low	Low
VanPetersen^19^	Netherlands	Radiological	228	Low	Low	High	Low	Low
Ferrari^20^	Italy	Radiological	60	High	Low	Low	Low	Low
Saad^21^	USA	Radiological	120	High	Low	High	Low	Low
Grabbe^15^	Germany	Radiological	340	High	Low	High	Low	Low
Ognjanović^1^	Serbia	Radiological	150	High	Low	High	Low	Low
Bertelli^22^	Italy	Radiological	1000	High	Low	High	Low	Low
Wickie^23^	Austria	Cadaveric	200	Low	Low	High	Low	Low
Wickie^23^	Austria	Radiological	80	High	Low	High	Low	Low

AQUA = Anatomical Quality Assessment.

### Statistical analysis

The statistical analysis was done using MetaXL version 5.3 (EpiGear International Ltd, Queensland, Australia). Two reviewers verified the analysis. Prevalence estimates for the AOB were assessed using a random effects model. Heterogeneity within the applicable studies was determined using the chi-squared and Higgins I-squared metrics^24^. A Cochrane’s Q p value of less than 0.10 was used as a threshold to indicate a significant level of heterogeneity. Four categories of interpretation for the Higgins I-squared thresholds were used: (1) between 0 and 40% was determined to be unimportant, (2) between 30 and 60% may be moderately important, 3) between 50 and 90% represents substantial heterogeneity, and 4) between 75 and 100% is considerable heterogeneity^24^. Modality, geographical distribution, gender, and laterality were taken into account using a subgroup analysis to determine sources of heterogeneity. Confidence intervals for each subgroup were compared to determine if statistically significant differences were found between each subgroup. Overlapping confidence intervals indicated no statistically significant differences between subgroups^24^. Figure 3 is a Forest Plot showing the mean and 95% confidence interval of the prevalence of the Arc of Buhler by study. The 95% confidence interval was arrived at using the Wilson Method for Confidence Intervals for Binomial Random Variables^25^.

## Results

### Quality assessment

Of the 11 studies that were included in this meta-analysis, there was a high risk of bias regarding the objectives and study characteristics for eight studies. The risk of bias associated with methodology characterization was also judged to be high in eight of 11 studies. The risk of bias within the study design, descriptive anatomy, and reporting of results was assessed as low for all 11 studies. The AQUA tool assessment for all studies can be found in Table 1.

### Study identification

**Figure 1:**
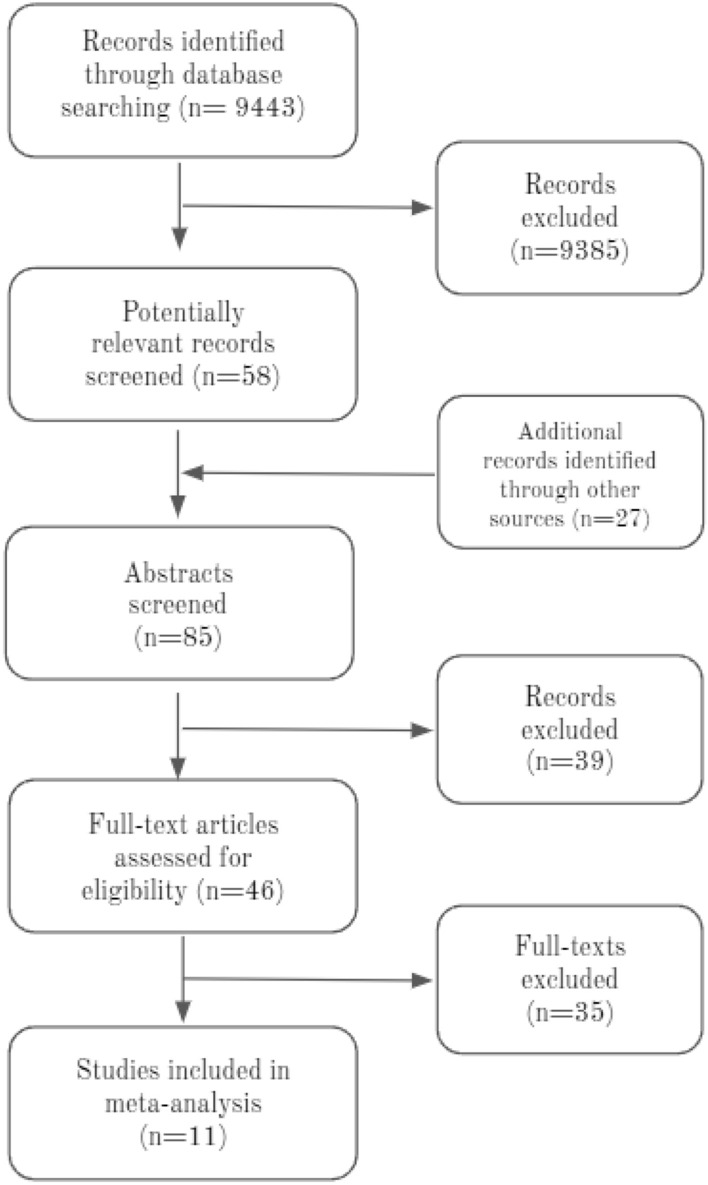
PRISMA flow chart showing the inclusion of studies within this meta-analysis.

The study identification process is summarized in Fig. 1. From a total of 9443 studies identified during the database searches, 58 articles were deemed to be potentially relevant according to the inclusion criteria. An additional 27 records were also identified during the reference search. After screening of abstracts and titles, 39 records were excluded (cases, case series, and irrelevant data). The full texts of the remaining 46 articles were analyzed. After review, 11 studies were ultimately included in this meta-analysis.

### Characteristics of the included studies

Characteristics of the included studies are summarized in Table 2. Of the 11 studies evaluated in this meta-analysis, radiological studies, including angiography and CT scans on live patients, dominated (n = 10). The other study relied on data from cadaveric specimens. The studies ranged in year from 1977 to 2016. Regarding geographic distribution of the included studies, eight were from Europe, one from North America, and two from Asia. Figure 4 shows a map of the countries of origin of the studies used in this analysis.

**Table 2:** Characteristics of included studies.

Study	Country	Type of study	Number of patients	Patients with AOB (%)
Farghadani^17^	Iran	Radiological (CT)	607	0.33
McNulty^6^	Ireland	Radiological (angiography)	300	1.00
Sureka^18^	New Delhi	Radiological (CT)	600	1.33
VanPetersen^19^	Netherlands	Radiological (angiography)	228	3.07
Ferrari^20^	Italy	Radiological (CT)	60	3.33
Saad^21^	USA	Radiological (angiography)	120	3.30
Grabbe^15^	Germany	Radiological (angiography)	340	4.12
Ognjanović^1^	Serbia	Radiological (CT)	150	2.67
Bertelli^22^	Italy	Radiological (angiography)	1000	0.30
Wickie^23^	Austria	Cadaveric	200	1.00
Wickie^23^	Austria	Radiological (angiography)	80	1.25

CT = computed tomography, AOB = arc of Buhler.

## Discussion

**Figure 2:**
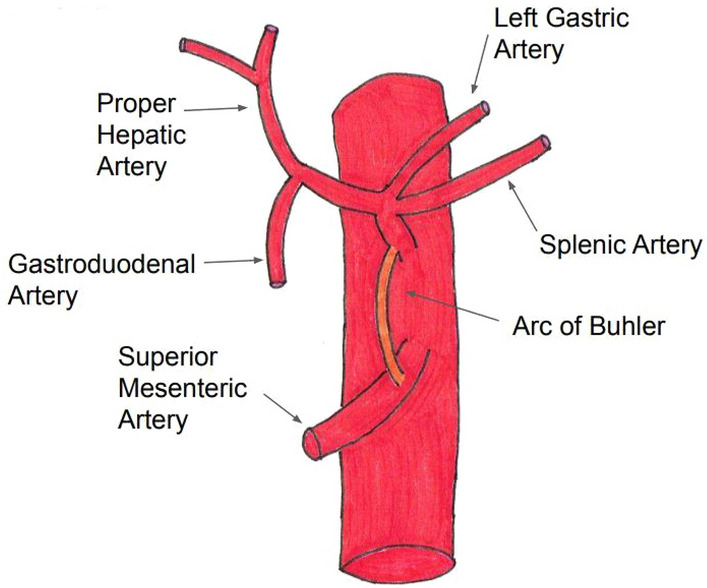
Diagram of the AOB.

The AOB usually directly connects the CAx and SMA in a retropancreatic course (Fig. 2), but it may also serve as an anastomosis between branches of either main vessel^8,9^. For instance, there are reports of anastomosis of the splenic artery with the SMA^26^, common hepatic artery with the SMA^27^, and CAx with the middle colic artery^28^.

Several additional variants can be found in the literature. In an estimated 1–2% of cases, the AOB can give off a branch to the pancreas^29^, and there may be anastomosis between the anterior and posterior pancreaticoduodenal arcades involving a branch of the dorsal pancreatic artery known as Kirk’s arcade^6^.

Saad et la. noted that, in all of the available cases (aside from cases of aneurysm) the AOB measured less than 2.5 mm in diameter, and half of them provided a hemodynamically significant collateral circulation^21^.

In general, the AOB is an incidental finding and is usually detected using imaging for unrelated abdominal issues^22^. This may raise concern that the sample may be skewed toward pathological cases, as cases appear retrospectively in the presence of other pathologies. It has been argued that the prevalence of the AOB is not sufficiently high to establish a relationship between a persistent AOB and stenosis of the CAx or SMA^4^; however, there is no positive evidence showing that an AOB is more common in individuals with occlusive disease than those with normal circulation^30^.

Knowing in advance whether a patient has an AOB is an important consideration for abdominal surgery and should be taken into account in preoperative planning, especially involving the regions of the pancreas, duodenum, and liver^16^. For example, in splenopancreatectomy, flow through the AOB may be reduced, and if this is not properly taken into account, it may result in increased risk of ischemia^29^. Appropriate planning for variant anastomoses has also been cited as crucial for planning in Whipple procedures^4^. Saad^21^ argued that it is of particular importance when dealing with prospective ligation of the gastroduodenal artery. It has been referred to as a rare cause of abdominal bleeding due to aneurysm, and may be a more significant risk in cases of CAx or SMA occlusion^21^.

The AOB is believed to be protective against ischemia in the event of compromised flow in the CAx or SMA, serving as a significant source of collateral circulation^30^. One case reported an AOB providing collateral blood flow in a patient suffering from median arcuate ligament syndrome in which a single vessel was occluded^11^. While the majority of findings are asymptomatic^21^, the pathology most directly associated with an AOB is aneurysm. Treatment of AOB aneurysm is most commonly performed by coil embolization^4^. There has been one instance of the AOB acting as a shunt, rather than a collateral, and diverting blood flow from the common hepatic artery; this was remedied by embolization of the AOB and restoring flow to the liver^31^. The AOB is also of interest to radiologists, as its detection using contrast may serve as helpful advanced knowledge for surgical procedures and may also have consequences for performing image-guided tumor therapy via catheter for hepatocellular carcinoma^30^.

**Figure 3:**
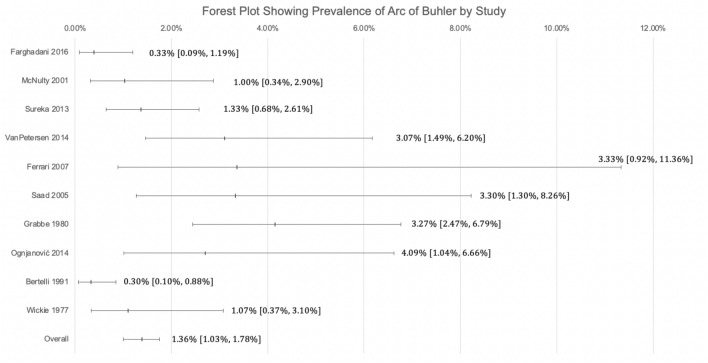
Forest Plot Showing the Prevalence of AOB by Study.

**Figure 4:**
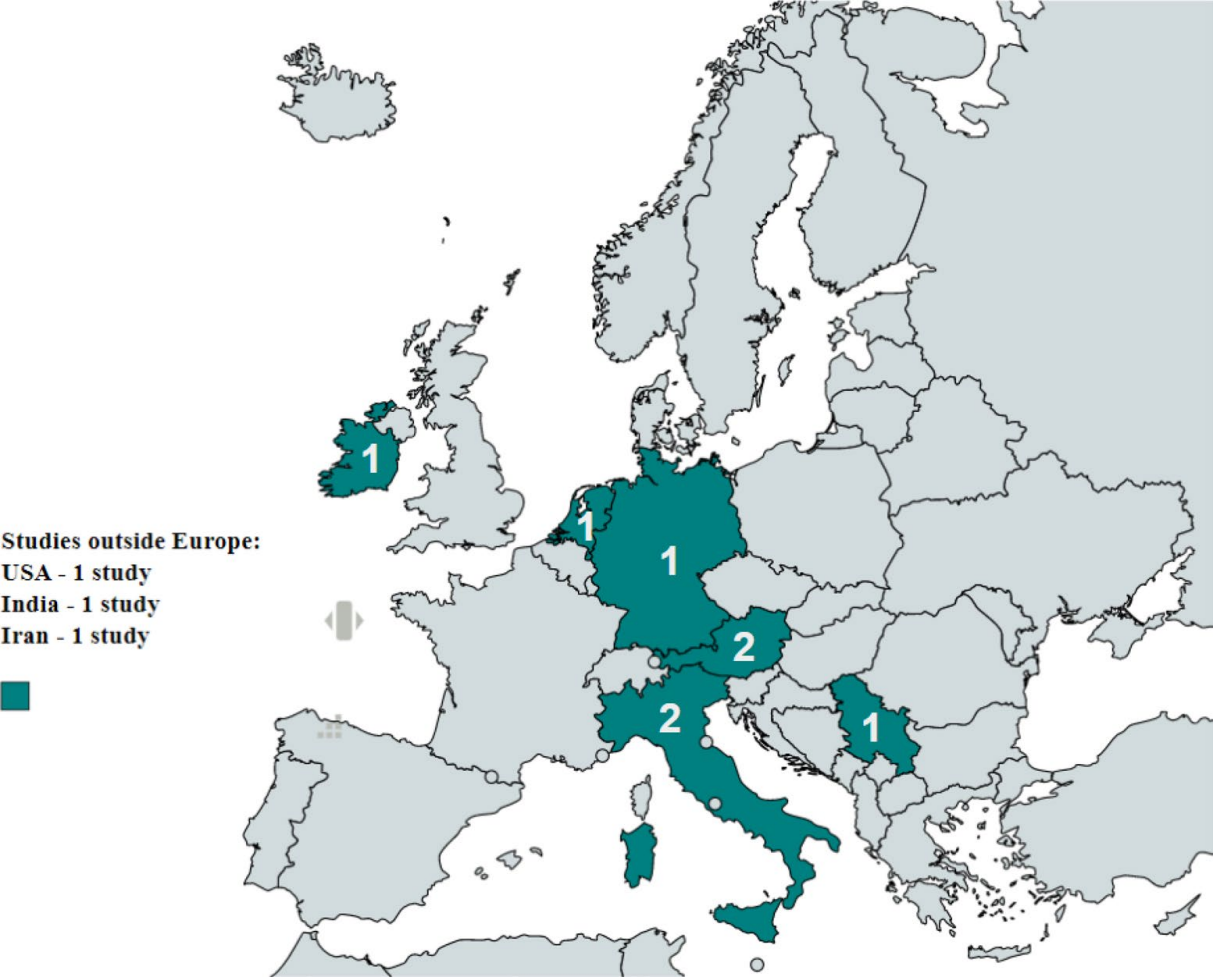
Map showing the locations of the studies used in this Meta-Analysis.

The main limitation of this study is the limited literature and high heterogeneity of many of the included studies as determined by the AQUA tool and confirmed by a funnel plot. The limited number and quality of existing studies indicates a need for more studies covering a large number of cases in order to provide a more accurate assessment of the prevalence of AOB and raise awareness among surgeons and radiologists (Figs. 3, 4).

## Conclusion

The AOB is an anastomosis of the CAx and the SMA or their branches. Prior to this study, it was estimated to have a prevalence in the range of 1–4% of the general population. Based on an extensive search of the literature, this study found that the best estimate of the overall pooled prevalence of the AOB among the general population is 1.7%, which falls within the expected range (Table 3). It is mostly asymptomatic, and the most highly associated pathology of the AOB is aneurysm, which is usually treated using coil embolization. The AOB is sufficiently significant to be considered during the planning phase of surgeries or radiological procedures involving the abdomen. Further interesting areas of investigation would be a more precise description of the embryology of the AOB as well as a more rigorous investigation of the relationship between the presence of the AOB and post-surgery symptoms of abdominal procedures.

**Table 3:** Prevalence of the AOB.

Subgroup	Number of studies (total number of patients)	Pooled prevalence of AOB: % (95% CI)	Level of heterogeneity determined using I-squared: % (95% CI)	Level of heterogeneity determined using Cochran’s Q: p-value
Overall*	11 studies (n = 3685)	1.7% (0.9, 2.9)	76.5% (57.9, 86.9)	*p* < 0.001
Radiological	10 studies (n = 3485)	1.8% (0.9, 3.0)	78.3% (60.5, 88.1)	*p* < 0.001
CT	4 studies (n = 1417)	1.4% (0.4, 3.0)	62.4% (20.6, 90.2)	*p* = 0.031
Angiography	6 studies (n = 2068)	1.9% (0.5, 4.0)	81.4% (67.0, 92.4)	*p* < 0.001

CT = computed tomography; CI = confidence interval.

*Includes cadaveric results.

## Data Availability

The datasets used and/or analyzed during the current study are available from the corresponding author upon reasonable request.
